# Hypersensitivity reactions to non beta-lactam antimicrobial agents, a statement of the WAO special committee on drug allergy

**DOI:** 10.1186/1939-4551-6-18

**Published:** 2013-10-31

**Authors:** Mario Sánchez-Borges, Bernard Thong, Miguel Blanca, Luis Felipe Chiaverini Ensina, Sandra González-Díaz, Paul A Greenberger, Edgardo Jares, Young-Koo Jee, Luciana Kase-Tanno, David Khan, Jung-Won Park, Werner Pichler, Antonino Romano, Maria José Torres Jaén

**Affiliations:** Allergy and Clinical Immunology Department, Centro Médico-Docente La Trinidad, Caracas, Venezuela; Department of Rheumatology, Allergy and Immunology, Tan Tock Seng Hospital, Singapore, Singapore; Allergy Service, Carlos Haya Hospital, Malaga, Spain; Department of Genetics and Environmental Risks, Faculty of Medicine, University of Lorraine, Lorraine, France; Allergy, Clinical Immunology and Rheumatology, Federal University of Sao Paulo, Sao Paulo, Brazil; Servicio de Alergia e Inmunología Clínica, Hospital Universitario, Monterrey, Nuevo Leon Mexico; Department of Medicine, Division of Allergy-Immunology, Northwestern University Feinberg School of Medicine, Chicago, IL USA; Immunology and Allergy Unit, Hospital Nacional Alejandro Posadas, Buenos Aires, Provincia de Buenos Aires Argentina; Department of Internal Medicine, Division of Allergy and Respiratory Medicine, Dankook University College of Medicine, Cheonan, South Korea; Department of Internal Medicine, Hospital das Clínicas, Clinical Immunology and Allergy Service, University of São Paulo, São Paulo, Brazil; Department of Allergy and Immunology, Hospital Servidor Público Estadual de São Paulo, São Paulo, Brazil; Department of Internal Medicine, Division of Allergy & Immunology, University of Texas Southwestern Medical Center, Dallas, TX USA; Department of Internal Medicine, Division of Allergy and Immunology, Yonsei University College of Medicine, Seoul, South Korea; Clinic for Rheumatology and Clinical Immunology/Allergology, Inselspital, Bern, CH 3010 Switzerland; Allergy Unit, Complesso Integrato Columbus, Rome, and IRCCS Oasi Maria SS, Troina, Italy

## Abstract

Antibiotics are used extensively in the treatment of various infections. Consequently, they can be considered among the most important agents involved in adverse reactions to drugs, including both allergic and non-allergic drug hypersensitivity [J Allergy Clin Immunol 113:832–836, 2004]. Most studies published to date deal mainly with reactions to the beta-lactam group, and information on hypersensitivity to each of the other antimicrobial agents is scarce. The present document has been produced by the Special Committee on Drug Allergy of the World Allergy Organization to present the most relevant information on the incidence, clinical manifestations, diagnosis, possible mechanisms, and management of hypersensitivity reactions to non beta-lactam antimicrobials for use by practitioners worldwide.

## Introduction

Adverse drug reactions (ADR) affect up to 10% of the population, and in hospitalized patients this figure increases up to 20% [1]. ADRs are classified into Type A (predictable), which comprises about 80% of all ADRs, and Type B (unpredictable). The predictable reactions include toxicity (overdose), side effects, secondary effects, and drug interactions, whereas the unpredictable reactions are intolerance, idiosyncracy, allergy and non-allergic hypersensitivity.

Unpredictable hypersensitivity reactions occur only in susceptible individuals, and can be produced through immunologic (allergic) or non-immunologic mechanisms [2]. Allergic reactions constitute 6 to 10% of all ADRs [3, 4]. Severe skin reactions such as Stevens-Johnson Syndrome (SJS), Toxic Epidermal Necrolysis, Drug-induced Hypersensitivity Syndrome (DiHS) and Acute Generalized Exanthematous Pustulosis (AGEP) are considered hypersensitivity reactions which can be life threatening and for which the pathophysiology is not completely understood. Patients presenting any of these reactions need prompt recognition to avoid lifelong sequels and cannot be re-exposed to the medication. No skin testing or desensitization protocols are available for these reactions, but patch testing may be helpful.

In the acute phase of an anaphylactic reaction, the elevated level of serum tryptase (1-4 hours after anaphylaxis) supports the diagnosis of drug hypersensitivity, IgE- or non IgE-mediated.

The two groups of drugs more often responsible for drug hypersensitivity reactions are antibiotics, especially beta-lactams, and non-steroidal anti-inflammatory drugs. Most publications on allergy to antibiotics have focused on hypersensitivity to penicillins and cephalosporins, while studies on reactions to each specific non beta-lactam are scarce or involve only case reports or a small series of patients. Since new antibiotics are continuously introduced into clinical use, reactions to newer compounds are likely to increase in the near future. The Special Committee on Drug Allergy of the World Allergy Organization organized a group of experts in this field to update the current knowledge on hypersensitivity reactions to non beta-lactam antimicrobials and produce a reference document that can be used worldwide by allergists and other practitioners. Some older antibiotics, which are currently in use less often, were included.

For each drug we will review the current data on the use of various in-vitro [5] and in-vivo diagnostic tests including skin prick tests (SPT) and intradermal tests (IDT) [6], measurement of specific IgE levels (immediate reactions), intradermal tests with delayed reading and patch tests (non-immediate reactions) [7] and drug provocation tests (DPT) (immediate and non-immediate reactions) [8]. The use of the basophil activation test (BAT) [9], lymphocyte transformation test (LTT) [10] and the ELISPOT test [11] for certain drugs will also be discussed. The utilization of some of these diagnostic methods has been described in several position papers [12, 13].

Other than avoidance of the putative drug, management of hypersensitivity reactions to non beta-lactam antimicrobials may also include tolerance induction or desensitization [14] where no alternative antibiotics can be used and the benefits of reintroducing the drug outweigh the risks. Protocols have been described for some of these medications.

## Aminoglycosides

### Introductory remarks

Aminoglycoside antibiotics have been used as an important part of the antibacterial drug arsenal for more than 50 years. They are indicated for polymicrobial and Gram negative bacillus infections. As the prevalence of aminoglycoside resistance has remained low, and emergence of bacterial resistance during therapy has been rare, they are still useful in clinical practice.

All aminoglycosides have an essential six-membered ring with amino group substituents — hence, the name aminocyclitol. The descriptor aminoglycoside results from the glycosidic bonds between the aminocyclitol and two or more amino-containing or non–amino-containing sugars. Aminoglycosides are classified in two groups: (A) streptidine group: e.g., streptomycin; (B) desoxystreptamine group: e.g., kanamycin, amikacin, gentamicin, tobramycin, neomycin. The most frequent and important side effects of the aminoglycosides are nephrotoxicity and ototoxicity. However, hypersensitivity reactions may occur [15].

### Epidemiology and risk factors

Neomycin and streptomycin induce allergic reactions in > 2% of treatments, gentamicin and amikacin in 0.1 to 2%, and kanamycin in 0.1 to 0.5%. No risk factors for allergy to aminoglycosides have been reported. The prevalence of allergic contact reactions to topical neomycin has been estimated between 1 and 29/10000 [16].

Neomycin is the most common sensitizer among topical medications [17]. Some geographical differences have been observed, since contact allergy to neomycin is much more prevalent in the United States (10-11.8%, mean 11.4%) than in Europe (1.2-5.4%, mean 2.6%) [18].

### Clinical manifestations

Contact dermatitis from topical aminoglycoside is the most frequent clinical manifestation associated with these antibiotics, since neomycin, gentamicin and tobramycin are widely used as cream, ointment, and eye or ear drops. The occurrence of positive patch test reactions to aminoglycosides increases with age in patients with chronic dermatosis [19]. Highest frequencies of sensitization to gentamicin have been found among patients with chronic otitis externa [20]. However, gentamicin has been regarded as less allergenic than neomycin [21]. It has been found that 30% of persons who have stasis ulcers, 15% of patients who have chronic otitis externa, and 5% of those who have various chronic eczematous conditions become sensitized by treatment with neomycin [22]. Caution must be taken in patients under systemic administration, when the drug can act as an internal allergen and reactivate eczema at a previously affected site [23].

Other cutaneous manifestations like urticaria, maculopapular rash, fixed drug eruption and toxic epidermal necrolysis (TEN) have been reported [15, 24].

As reported recently, a patient developed a drug reaction with eosinophilia and systemic syndrome (DiHS, DRESS) induced by amikacin used for the treatment of septic arthritis of the knee, which was confirmed by patch tests and immunobiologic tests. Cross-reactivity with other aminoglycosides was not observed in this patient [25].

Anaphylaxis is very uncommon. Few anaphylactic reactions to streptomycin have been reported in which the drug was present in contaminated food, or added to cell culture media and administered during *in vitro* fertilization, or during immunotherapy with phytohemagglutinin-lymphokine-activated killer cells, or absorbed through skin lesions of a subject affected by hand allergic contact dermatitis. In most of these reactions an IgE-mediated mechanism was suspected on the basis of skin-test positivity [25]. Gentamicin-induced anaphylaxis is rarely reported. In one case report a positive prick test to gentamicin was found and positive patch tests not just to gentamycin but also to other aminoglycosides suggested a combined type I/type IV sensitization [26, 27].

Also there have been reports of adverse reactions to inhaled tobramycin, including persistent eosinophilia with severe bronchospasm [28] and cutaneous rash [29].

### Pathogenesis

There is no definitive evidence of IgE-mediated immediate hypersensitivity to aminoglycosides. IgG anti-streptomycin antibodies have been demonstrated in patients with haemolytic anemia. Contact dermatitis is mediated by cell-mediated delayed hypersensitivity, and can be demonstrated by means of patch testing. It is known that neomycin is one of the most potent and frequent contact sensitizers producing contact allergy all over the world. Neomycin-induced contact dermatitis occurs especially in patients with leg ulcers, atopic eczema, or chronic conjunctivitis or otitis, and in patients with long-term cutaneous use of the drug [30]. Other systemic manifestations such as eosinophilia, bronchospasm and serum sickness are rarely observed.

### Diagnosis

There is no validated skin test for the diagnosis of immediate hypersensitivity to aminoglycosides. Positive tests have been observed with tobramycin, gentamicin, framycetin and streptomycin [26, 27, 31, 32]. However, a cautious approach must be taken when evaluating anaphylactic reactions to streptomycin, since systemic reactions have been observed after prick test. The starting concentrations suggested for prick tests range from 0,1 to 1 ng/mL, gradually reaching the concentration of 20 mg/mL if needed. If prick tests are negative intradermal testing can be performed and non-irritating concentrations for intradermal testing have been established for gentamicin and tobramycin to be 4 mg/mL [33]. There is no evidence of positive serum IgE to aminoglycosides [26].

Patch tests with reading at 72 and 96 hours are recommended for the diagnosis of non-immediate reactions. The concentration for neomycin, gentamicin and tobramycin is 20% in petrolatum, and 1% for streptomycin [34]. The percentage of positive patch tests with neomycin in patients with contact dermatitis is 2.5 to 3.6%, and in patients with leg ulcers it varies from 9 to 15%. However, some patient series show higher prevalences of sensitization [35]. Patch tests with neomycin sulfate are positive in 5% of children with contact dermatitis younger than 3 years [36].

Tests by prick, intradermal, intramuscular, or subcutaneous routes have not been standardized. Interferon-γ ELISPOT has been recently utilized for the diagnosis of amikacin-induced DIHS [21].

### Management

Aminoglycosides should be avoided in patients with a diagnosis of hypersensitivity. Cross-reactions among aminoglycosides are common in patients with contact dermatitis, approaching to 50% or more between those from the desoxystreptamine group. Cross-reactivity is less common to streptomycin (1-5%) [32]. However, there are reports of eczematous contact-type dermatitis after systemic administration of streptomycin in individuals who had become sensitized to neomycin and had never been exposed to streptomycin [22]. Cross-reactivity between neomycin, sisomycin and amikacin is 20%, and between neomycin, netilmycin and streptomycin 1 to 5% [21, 29].

Streptomycin shows no cross-reactivity with other aminoglycosides that share deoxystreptamine [37], or with those that are disubstituted-4,5 (neomycin and paromomycin), which show high cross reactivity with each other, nor disubstituted-4,6 ones (tobramycin, kanamycin, amikacin, gentamicin) whose reactivity with neomycin is variable but always low at around 50% [32]. Some experts recommend avoidance of all aminoglycosides in neomycin-sensitive patients.

Desensitization is possible by the intravenous route in patients with urticaria or angioedema due to streptomycin [38, 39] and for tobramycin both intravenously and via inhalational route [40].

## Chloramphenicol

### Introductory remarks

This antibiotic produced by *Streptomyces venezuelae* contains a nitrobenzene ring linked to propanol, with an amide group binding to a derivative of dichloroacetamide acid. Chloramphenicol is bacteriostatic against Gram positive anaerobic and Gram negative aerobic and anaerobic bacteria. Presently it is uncommonly used due to the risk of hematologic adverse effects.

### Epidemiology and risk factors

Hematological effects of chloramphenicol are idiosyncratic effects, non-immunologically mediated. Generally speaking, allergy to chloramphenicol is uncommon. However, contact dermatitis can occur in up to 12-13.9% of patients with venous leg ulcers [41, 42]. Other risk factors include allergy to penicillin or ampicillin [43], severe infection, and previous exposure to phenicols.

### Clinical manifestations

The following adverse reactions have been observed: Systemic reactions (anaphylactic shock [44, 45], fever), cutaneous symptoms (urticaria [46], angioedema, maculopapular rash, acute generalized exanthematous pustulosis [47], contact dermatitis [48, 49], bullous eruption, erythema multiforme, exanthemas, fixed drug eruption, SJS, TEN, respiratory symptoms (bronchospasm), hematologic manifestations (aplastic anemia [in 1 out of 21600 treatments], and reduction of erythrocyte counts).

### Pathogenesis

The mechanisms of reactions to chloramphenicol are unknown. It is likely that the dichloroacetamide ring is the major antigenic determinant.

### Diagnosis

Skin prick tests and patch tests (chloramphenicol 1% in petrolatum) have been proposed. Specific IgE in the serum is not clinically relevant.

### Management

Avoidance of chloramphenicol and cross-reacting synthetic derivatives is recommended.

## Clindamycin

### Introductory remarks

Clindamycin is a chemical derivative of lincomycin with activity against aerobic Gram positive and anaerobic Gram negative bacteria. Adverse effects of clindamycin include diarrhea, pseudomembranous colitis, metallic taste in the mouth, transient elevations in liver transaminases, granulocytopenia and thrombocytopenia. Hypersensitivity reactions have decreased in frequency and are relatively uncommon.

### Epidemiology and risk factors

Clinical studies from the 1970s reported an incidence of delayed rashes of approximately 10% with the use of clindamycin [50, 51]. A much larger study of 3,896 clindamycin administrations from a single U.S. hospital reported an incidence of < 1% of adverse drug reactions with only 5 probable cutaneous reactions in 3,462 patients, none of which were severe [52]. Risk factors for clindamycin allergic reactions are unknown.

### Clinical manifestations

The most common presentation for clindamycin allergy is a delayed maculopapular exanthem, usually 7-10 days after initiation of the drug [53]. However, other immunologic drug reactions have been reported including anaphylactic shock, urticaria, angioedema, fixed drug eruptions, bullous eruptions, AGEP, Sweet’s Syndrome, SJS, and DiHS/Drug Rash with Eosinophilia and Systemic Symptoms (DRESS) [54–61].

### Pathogenesis

The pathogenesis of the most common form of clindamycin allergic reaction, delayed maculopapular exanthems (MPE), has not been well studied. Patch tests have been shown to be positive suggesting that these reactions may involve T-cell mediated hypersensitivity [53].

### Diagnosis

Skin prick tests (SPT) and intradermal tests (IDT) with clindamycin have not been found to be useful for diagnosis. In a study of 31 subjects with histories suggestive of immunologically mediated reactions, all patients were subjected to prick and intradermal testing followed by oral challenge. None of the patients had a positive prick or intradermal test [62]. However, ten of 31 patients (31%) had a positive oral challenge.

Patch testing with clindamycin has yielded mixed results with positive tests ranging between 15-30%. A study from Germany of patients with a history suggestive of clindamycin skin reactions found that 5/33 patients (15%) had positive clindamycin patch tests using pulverized 150 mg tablets in 1 mL saline [63]. False negative patch tests were seen in 6/26 patients. Oral challenges using hourly dosing of 75, 150, 300, and 450 mg of clindamycin were performed and 6 of 26 subjects had positive challenges, all showing exclusively cutaneous manifestations. Another study from Portugal of 30 patients with delayed cutaneous reactions associated with clindamycin, found positive patch tests in 30% of patients using clindamycin 10% in petrolatum [53]. Oral challenges with clindamycin were not performed in this study. Given the potential for false negative reactions on patch testing, oral challenge is required to confirm tolerance to clindamycin.

### Management

Most clindamycin delayed maculopapular exanthems do not require specific therapy and resolve spontaneously with cessation of the drug. A single case report of successful drug desensitization to clindamycin in a patient with HIV infection has been published [64]. This patient had a delayed generalized exanthem to clindamycin, confirmed by subsequent challenge. An induction of drug desensitization was performed starting with a dose of 20 mg every 8 hours followed by dose escalation daily with 40, 80, 150, 300, then 600 mg over 6 days.

## Dapsone

### Introductory remarks

Dapsone is a sulfone antimicrobial that is a principal drug in a multi-drug regimen recommended to treat leprosy. Other uses as an antimicrobial include treatment of malaria and *Pneumocystis jiroveci* pneumonia. Dapsone also has anti-inflammatory effects and has been used to treat dermatitis herpetiformis and a wide variety of other inflammatory dermatological conditions including urticaria. Dapsone may cause a variety of adverse effects that may be categorized as pharmacologic, allergic, or idiosyncratic.

### Epidemiology and risk factors

Pharmacological hematologic adverse effects of dapsone are common and dose-dependent. Patients with glucose-6-phosphate dehydrogenase or glutathione reductase deficiency are more susceptible to these hematologic effects. Asymptomatic, clinically insignificant methemoglobinemia occurs in most patients on dapsone at a dose of 100 mg daily [65]. A dose-related hemolysis occurs in 4% of patients with HIV infection and in patients with stem cell transplantation a frequency of hemolysis as high as 87% is observed [66, 67]. Hemolysis at doses of 100 mg daily is generally mild and even in the stem cell transplant cohort did not require cessation of dapsone. Agranulocytosis is a rare idiosyncratic reaction associated with dapsone and occurs in 1:10,000-20,000 patients not otherwise ill taking it as an antimalarial agent [68]. Patients with dermatitis herpetiformis have a much higher risk of agranulocytosis with an estimated risk 25-33 fold higher than normal individuals.

The most common hypersensitivity reaction to dapsone is a generalized exanthem. The incidence of this rash varies widely in different reports and by underlying disease being treated. A large study of 521 HIV patients treated with dapsone 100 mg/d reported that 18% had “hypersensitivity” reactions although no further details were presented [69]. A retrospective study of 75 HIV patients treated with dapsone found that 16% reported a rash but after a critical evaluation of each case, only 2 cases (3%) were judged as “likely related” to dapsone [66]. In contrast, in a study of 233 leprosy patients treated with dapsone doses of 50-100 mg daily for 3 years none of them were required to stop the treatment because of undesirable side effects and the drug was well tolerated [70].

The most serious hypersensitivity reaction to dapsone is known as the dapsone hypersensitivity syndrome which is likely a form of DiHS/DRESS. A recent systematic review calculated an incidence of dapsone hypersensitivity syndrome of 1.4% with an overall case-fatality of 9.9% [71]. Risk factors for fatalities from dapsone hypersensitivity syndrome include mucosal involvement, rash, hepatitis, older age, leprosy as an indication for dapsone, and living in non-affluent countries.

### Clinical manifestations

Methemoglobinemia with levels under 20% is usually asymptomatic. Dyspnea, nausea, tachycardia and cyanosis occur at higher levels and mental status changes, seizures and arrhythmias occur with methemoglobin levels > 50%. Hemolysis from dapsone is typically asymptomatic but with more severe anemia patients may present with dyspnea on exertion and fatigue. Agranulocytosis typically presents acutely as fever and evidence of bacterial infection.

Maculopapular exanthem is the most common hypersensitivity reaction to dapsone and is similar to other drug reactions. Dapsone hypersensitivity syndrome has a mean latency of 4 weeks prior to symptoms. A systematic review of the dapsone hypersensitivity syndrome reported the following frequency of signs and symptoms: fever (96%), rash (92%), hepatitis (82%), lymphadenopathy (74%), nausea and vomiting (61%), eosinophilia (45%) and mucosal involvement (45%) [71].

Other less common manifestations of dapsone adverse reactions include photodermatitis, eosinophilic pneumonia, pancreatitis, hepatitis and SJS/TEN [72–75].

### Pathogenesis

The common hematologic adverse effects of dapsone (methemoglobinemia and hemolysis) are due to its hydroxylamine metabolite. Dapsone hydroxylamine reacts with oxyhemoglobin (Fe2+) to form methemoglobin (Fe3+) [65] Dapsone-induced hemolysis is thought to involve the generation of free radicals by dapsone hydroxylamine and subsequent depletion of red blood cell (RBC) glutathione stores [67]. The mechanism of dapsone induced agranulocytosis is unknown and has been speculated to involve cell control mechanisms as opposed to toxicity or an immunologic reaction [68].

The mechanism of the dapsone hypersensitivity syndrome is unclear [76]. It is unknown whether dapsone hydroxylamine metabolites are causative of dapsone hypersensitivity syndrome. Circulating autoantibodies have been reported after dapsone hypersensitivity but the pathogenic relevance of these antibodies is unknown [77].

### Diagnosis

Dapsone-induced methemoglobinemia can be diagnosed by a methemoglobin level measurement. Levels <15-20% are usually asymptomatic; however in patients with anemia, significant cardiac or respiratory diseases, or other hemoglobin abnormalities, symptoms may occur with levels < 15% [78]. Dapsone-induced hemolysis can be detected via peripheral blood smear by the presence of numerous bite cells or eccentrocytes [77]. Significant hemolysis may be detected by measuring hemoglobin, lactate dehydrogenase, indirect bilirubin and haptoglobin levels. Agranulocytosis can be detected by loss of peripheral granulocytes on a complete blood count with differential counts.

There are no well-established criteria for the diagnosis of dapsone hypersensitivity syndrome. However, as this reaction is most likely a form of DiHS/DRESS, using the criteria for diagnosis of DIHS/DRESS is appropriate [79, 80]. Specific drug diagnostic tests such as patch testing have not been well studied for dapsone syndrome.

In a retrospective study, cross-reactivity between dapsone and trimethoprim-sulfamethoxazole was observed in 13 out of 60 HIV-infected patients (21.7%) [81].

### Management

Symptomatic methemoglobinemia from dapsone is typically treated with methylene blue. Cimetidine has also been reported to be effective at reducing methemoglobinemia in most case series [78]. Vitamin E has been reported to be partially protective against dapsone-induced hemolysis [82]. Management of agranulocytosis from dapsone involves stopping the drug and treating any underlying infections.

Drug desensitization procedures have been reported for dapsone since 1963. The largest and oldest case series described 52 leprosy patients with a history of a generalized papular dermatitis precipitated by dapsone, some of whom likely had dapsone hypersensitivity syndrome [83]. An induction of drug tolerance procedure has been reported starting with 12.5 mg dapsone twice a week with gradual dose escalation until a dose of 100 mg was reached in 15 weeks. Forty-eight patients were successfully made tolerant; however, 26 patients had recurrence of dermatitis during the procedure (1-10 times) before a tolerant dose was maintained. Another case series in 14 HIV patients with fever and rash from dapsone underwent an induction of drug tolerance procedure over 42 days starting with daily doses of 0.01 mg [84]. All but one patient achieved drug tolerance to dapsone. Finally, a case of a patient with dermatitis herpetiformis with dapsone hypersensitivity syndrome underwent a gradual reintroduction of dapsone starting at 50 mg (the last tolerated dose) and increased the dose gradually to 100 mg twice daily over 18 weeks with tolerance of the therapeutic dose [85].

## Ethambutol

### Introductory remarks

Ethambutol is one of the first-line drugs for the treatment of active mycobacterial tuberculosis (TB) infection, and is most commonly used in combination with other drugs such as isoniazid, rifampicin, and pyrazinamide [86].

Although ethambutol is generally well tolerated, ADRs including allergic reactions to this drug can occur. Of those adverse reactions, optic neuritis is the main and most frequently recognized. Serious hypersensitivity reactions, serious cutaneous and hematological reactions have also been reported.

### Epidemiology and risk factors

First-line anti-tuberculosis drugs are associated with significant ADRs [87]. According to a clinical-based study, female sex, old age, Asian ancestry and human immunodeficiency virus (HIV) infection have been suggested to be associated with an increased incidence of reactions to first-line anti-tuberculosis medications [88]. Among 430 patients, the incidence of all major adverse effects was 0.07 per 100 person-months of exposure for ethambutol (95% CI 0.04– 0.10), which means adverse reactions to ethambutol were uncommon compared to the other anti-TB drugs [88].

Ethambutol is relatively safe compared to the other anti-TB drugs and a prevalence of less than 1% of optic neuritis has been reported in patients who received a 15 mg/kg dose [89]. Dermatologic reactions are relatively uncommon and skin rash occurred in 0.15% [86]. Serious allergic reactions to ethambutol are only reported as case series, suggesting a low incidence.

### Clinical manifestations

Common reactions reported from field trials of first line anti-TB drugs include skin rash and pruritus, hepatitis, nausea/vomiting, thrombocytopenia, influenza-like illness, arthralgias and neuropsychiatric symptoms [89].

However, most of these ADRs are usually induced by anti-tuberculosis drugs other than ethambutol. The most common problematic adverse reaction induced by ethambutol is optic neuritis, which appears to be dose related and may cause decreases in visual acuity, color blindness, and irreversible blindness. The changes in visual acuity may be unilateral or bilateral.

Ethambutol-induced skin reactions consist of hair loss, rash, pruritus, urticaria, angioedema, skin striae, and exfoliative dermatitis [90]. Serious cutaneous adverse reactions such as erythema multiforme, SJS, and TEN have also been reported [91, 92]. Skin rash, blood eosinophilia and pulmonary infiltrates can rarely be observed [93]. There are case reports of DiHS/DRESS induced by ethambutol [94, 95]. Other adverse effects of ethambutol include gastrointestinal intolerance, hyperuricemia, peripheral neuropathy, and hematologic changes.

### Pathogenesis

No studies on the pathogenesis of ethambutol-induced allergic reactions have yet been reported. Optic neuritis develops as a result of demyelination and not from an inflammatory process. Recently, the association of HLA markers with anti-TB drug-induced DiHS syndrome has been published, suggesting a possible immunological involvement [96].

### Diagnosis

There is no gold standard test for the diagnosis of ethambutol-induced allergic reactions. Oral provocation test is not recommended when serious reactions occur. Skin tests are usually negative. Patch tests may give valuable information.

Clinical information is important when trying to decide the culprit drug causing an allergic reaction. However, culprit drug(s) for these adverse reactions are usually not clear and the assessment of a cause-effect relationship is not easy because they are seldom used alone. Allergic reactions to combination therapy of the anti-TB drugs may be due to drug allergy to more than one culprit drug.

### Management

Testing of visual acuity before administration of ethambutol and regular monitoring during drug treatment are necessary for the early detection of optic neuritis. Monthly evaluation of visual acuity is recommended when doses higher than 15 mg/kg are administrated. Decreased visual acuity usually recovers over a period of weeks to months after ethambutol discontinuation, although occasionally visual impairment and even blindness can be permanent.

When serious allergic reactions occur, all the anti-TB drugs should be promptly discontinued. With improvement after discontinuation of all anti-TB drugs, sequential re-challenge is usually done cautiously, starting with a small dose. When ethambutol is confirmed as the culprit drug, anti-TB drug combinations without ethambutol can be administered for the treatment of tuberculosis. Rapid desensitization has been utilized successfully in 5 patients with various ethambutol reactions [97].

## Isoniazid

### Introductory remarks

Isoniazid is a component of first-line treatment for active TB and is administered as well for treatment of latent TB [98, 99]. Well recognized adverse effects from isoniazid include elevation of liver enzymes, drug fever, peripheral neuropathy, pruritus, and very infrequently a rash. SJS has been described [100]. There is a single report of anaphylaxis [101]. Isoniazid inhibits both diamine oxidase and monoamine oxidase, thus allergic-like reactions can occur when foods containing histamine or tyramine are ingested [102].

### Epidemiology and risk factors

In 10-20% of patients receiving first-line medications for TB disease, it can be anticipated that there will be asymptomatic, typically reversible elevations up to at least 5 times the upper limit of normal for alanine aminotransferase (ALT) and aspartate aminotransferase (AST). The incidence of clinical hepatitis ranges from 0.1% to 0.6% [99]. This incidence increases to approximately 1.6% if isoniazid and other medications excluding rifampicin are administered. Clinical hepatitis is reported to occur in 2.7% of patients receiving both isoniazid and rifampicin. Risk factors include age (50-64 years), underlying liver disease, especially associated with alcohol, the post-partum period, and being a Hispanic woman. Deaths from clinical hepatitis occur in less than 1/5000 patients. Peripheral neuropathy is considered dose related with an incidence < 0.2% [100]. Risk factors include nutritional deficiency (reason for supplementation with vitamin B6, pyridoxine, 25 mg daily), alcoholism, diabetes mellitus, human immunodeficiency virus (HIV) infection/acquired immunodeficiency syndrome (AIDS), renal failure, pregnancy and lactation. As many as 20% of patients develop anti-nuclear antibodies, but systemic lupus erythematosus that involves discontinuation of isoniazid occurs in < 1% of patients [99].

The precise incidence of SJS/TEN from isoniazid has not been established, although it seems to be rare. HIV infection is well recognized as a risk factor for drug hypersensitivity. In a series of 820 patients undergoing observed therapy for TB Disease, 47 patients (5.7%) developed cutaneous adverse reactions, with pyrazinamide being the most likely drug [103]. Isoniazid-associated cutaneous reactions were noted in 0.98% of patients [103].

### Clinical manifestations

Allergic reactions to isoniazid include rashes that are morbilliform or lichenoid (violaceous, flat topped, pruritic papules), flushing, and extremely rarely, SJS/DiHS/DRESS [99, 100]. In a study where incriminated anti-TB medications were re-administered to patients having experienced cutaneous adverse drug reactions, often beginning at 1/8 of the total dose, reactions to isoniazid were reproducible in 5/36 (13.8%) patients [100]. In this series, the 5 reproduced reactions were SJS, DiHS and lichenoid rash, typically within the first 72 hours. For comparison, reproducible reactions occurred with rifampicin in 13/37 (35%) patients, and with pyrazinamide in 3/15 (20%) patients. These data emphasize that in patients receiving multiple medications for TB disease and in whom medications for HIV/AIDS may be administered concurrently, it is often difficult to ascertain the exact putative drug. Frequently, patients receiving isoniazid experience a rash in the first month of therapy (median onset at 20 days). In a series of gold miners receiving treatment for latent TB, cutaneous rashes occurred in 61 patients (0.25%), most of which were considered mild or moderate in severity. Pruritus was reported in the first month by 4.3% of gold miners being treated for latent TB, and by 5.3% of patients when combining symptoms from the visits at 3 and 6 months of treatment [104]. For comparison, the authors noted that pruritus had been reported by 8.3% of patients in the 2 week period prior to initiation of isoniazid. These data do suggest that the benefits of continuing isoniazid in patients with pruritus outweighs the risks of treatment, but careful monitoring for the presence of rash is needed.

Drug fever from isoniazid ranges from 38-40°C and by definition resolves within 72 hours of discontinuation of the incriminated medication [105]. Peripheral neuropathy is characteristically mild, but patients with more than mild preexisting peripheral neuropathy should not receive treatment with isoniazid [104].

### Pathogenesis

Evidence for isoniazid hypersensitivity and toxicity reactions includes studies from hepatitis, hepatic necrosis and drug induced lupus erythematosus. One notion is based on the metabolism of isoniazid to reactive or immunogenic molecules. Initially, isoniazid is acetylated by N-acetyltransferase 2 to acetylisoniazid and then hydrolyzed to acetylhydrazine, which then is oxidized to highly reactive metabolites that acetylate various molecules [106]. The acetylated molecules damage cells directly or form immunogenic protein complexes that result in tissue injury. It had been proposed that the rapid acetylator of isoniazid phenotype would be protected from hepatic damage. In studying both the genetics of N-acetyltransferase 2 and the pharmacokinetics of acetylation by determining the ratio of serum acetylisoniazid/isoniazid after ingestion of 300-400 mg of isoniazid, the functional contribution of genetic polymorphisms and acetylation phenotypes could be elucidated more precisely [107]. In patients who developed hepatitis, some polymorphisms of N-acetyltransferase 2 were associated with slow acetylation (higher serum concentrations of isoniazid and lower ratios of acetylisoniazid/isoniazid). Other genetic mutations in drug-metabolism were not associated with isoniazid hepatitis. Continued research is required as not all rapid acetylator patients are protected from hepatitis.

### Diagnosis

Allergic drug reactions typically follow the “rule of 2 s” in that most reactions occur in the first 2 minutes to 2 months after initiating therapy [108]. In the setting of possible isoniazid associated cutaneous reactions, other medications used for TB or HIV/AIDS may be the cause as opposed to isoniazid. Since specific skin tests or *in vitro* tests are often not available to confirm isoniazid as the culprit, in some settings, such as treatment of TB disease or for treatment of latent TB, graded challenges may be indicated to confirm or, more preferably, exclude drug hypersensitivity.

Non-allergic reactions such as elevations in hepatic transaminases do not necessitate discontinuation of isoniazid unless clinical hepatitis or more than 5-fold increases over the upper limits of normal occur. Clinical judgment is required as other host factors can increase the risk of isoniazid hepatotoxicity.

### Management

As noted above, some patients with pruritus associated with initiation of isoniazid can have their therapy continued in the absence of a new rash. Even patients with morbilliform rashes may receive concomitant pharmacotherapy (histamine 1 receptor antagonists, topical anti-pruritus treatments and topical corticosteroids) for symptom relief so as to be able to continue the essential isoniazid. Often, a more serious rash that is generalized, erythematous, raised and potentially blistering, would result from another medication (or viral infection) rather than from isoniazid. Consultation with an allergist-immunologist to help prioritize the potential culprit medications or other causes of the new rash is recommended where feasible. Graded challenges or desensitizations with isoniazid may be required, and various protocols can be utilized. Starting doses in adults have been at 40-50 mg, but sometimes lower doses may be advisable. Daily increments, such as reaching 300 mg in 3-7 days have been reported [100, 109].

The patient and referring physician or health care professional need to accept the possibility that a serious cutaneous reaction that requires oral or intravenous corticosteroids could occur during or after the graded challenge. If the possibility of future systemic corticosteroids is refused, the graded challenge should not be undertaken. Fortunately, most graded challenges with isoniazid would be expected to be carried out safely and with a favorable benefit-risk ratio.

## Macrolides

### Introductory remarks

Macrolides are classified according to the number of carbon atoms in their lactone ring: 14-membered (erythromycin, troleandromycin, roxithromycin, dirithromycin, and clarithromycin), 15-membered (azithromycin), and 16-membered (spiramycin, rokitamycin, josamycin, and midecamycin). Macrolides exhibit a good activity against Gram-positive aerobes and some Gram-negative aerobes.

### Epidemiology and risk factors

Hypersensitivity reactions to macrolides are relatively uncommon (0.4% to 3% of treatments) [110].

### Clinical manifestations

Cases of immediate reactions in the form of urticaria/angioedema, rhinoconjunctivitis, and anaphylaxis, and nonimmediate reactions, such as maculopapular rash, non-immediate urticaria, contact dermatitis, fixed drug eruptions, and toxic epidermal necrolysis, have been reported in children and adults [110–115].

### Diagnosis

A study by Empedrad et al [33] on skin testing found non-irritating concentrations for intradermal testing of erythromycin (0.05 mg/mL) and azithromycin (0.01 mg/mL). However, data from the literature indicate that in evaluating hypersensitivity reactions to macrolides, the sensitivity of skin tests is low; therefore, provocation tests are often necessary [110, 112, 114]. Specifically, Seitz et al evaluated 125 subjects with suspected macrolide allergy. IDT with erythromycin, clarithromycin, and azithromycin were performed at a concentration of 0.01 mg/mL. All skin tests were negative in the 53 patients with immediate reactions, whereas one of the 72 subjects with non-immediate reactions developed a delayed SPT positivity to roxithromycin at 50 mg/mL. Challenges were negative in the 47 subjects with immediate reactions who underwent such tests, whereas they were positive in 4 of 66 patients with non-immediate reactions [115]. A study by Mori et al [114] evaluated 64 children with histories of clarithromycin hypersensitivity by performing intradermal tests at a concentration of 0.5 mg/mL, and they subsequently underwent challenges. Intradermal test sensitivity and specificity were 75% and 90%, respectively.

In single cases, skin tests proved to be useful in diagnosing IgE-mediated hypersensitivity to macrolides such as erythromycin, spiramycin, azithromycin, and roxithromycin [110, 116–118].

There are also reports of positive responses to patch tests at concentrations up to 10% in petrolatum or dimethylsulfoxide in subjects with non-immediate reactions (e.g., fixed drug eruptions and contact dermatitis) to macrolides such as erythromycin and azithromycin [110, 111, 113].

With regard to *in vitro* tests, there are reports of positive serum specific IgE assays in single cases [116], while in the study by Seitz et al [115], Basophil activation Test (BAT) and Lymphocyte Transformation Test (LTT) were negative in 10 and 7 subjects, respectively.

### Management

As far as the management of subjects with macrolide hypersensitivity is concerned, cross-reactivity among 14-membered macrolides (erythromycin, clarithromycin, and roxithromycin) has been detected in single patients with either immediate [117] or non-immediate [111] reactions to erythromycin on the basis of positive responses to prick tests or patch tests. Milkovic-Kraus et al described two subjects with allergic contact dermatitis to azithromycin who showed cross-reactivity with azithromycin intermediates, including erythromycin [113]. However, the paucity of reports of allergic contact dermatitis to azithromycin makes it difficult to advise avoidance of other macrolides. In any case, it would appear that macrolide hypersensitivity is unlikely to be cross reactive. Desensitization has been successful in a few cases of macrolide hypersensitivity [110, 118].

## Pyrazinamide

### Introductory remarks

Pyrazinamide (PZA), a synthetic analog of nicotinamide, is one of the most effective antituberculous drugs.

### Epidemiology and risk factors

Hypersensitivity reactions to anti-TB drugs are reported in 1-5% of patients. Among 430 patients, the incidence of all major adverse effects was 1.48 per 100 person-months of exposure (95% CI 1.31-1.61) for pyrazinamide, which was the highest risk among other first line anti-TB medications [88]; cutaneous manifestations are the most frequent, and PZA has been involved in most such cases as it is often used in combination with isoniazid, rifampicin and ethambutol in the initial treatment of TB [119]. Pyrazinamide-induced adverse events were associated with patients aged over 60 and born in Asia.

### Clinical manifestations

It is difficult to identify the responsible compound in subjects treated simultaneously with 4 different antituberculous drugs. In particular, PZA may cause hypersensitivity reactions, such as flushing, immediate (i.e., occurring within one hour after the last drug administration) itchy rashes [120–125], and anaphylaxis [126, 127]. PZA can also provoke dose-dependent cytolytic hepatitis.

### Pathogenesis

With regard to the pathogenic mechanisms of these reactions, considering that nicotinamide – from which PZA is synthesized regularly – can cause truncal and facial flushing and itching, presumably prostaglandin-mediated, a similar mechanism has been hypothesized for PZA-induced flushing and skin rash [126]. Moreover, Soyez et al observed an increase of plasma histamine levels on the first day of a desensitization protocol applied in a subject who had reacted to PZA [120]. In the case report by Shorr and Trotta [121], however, biopsy of the rash revealed a spongiotic dermatitis with eosinophils and necrotic keratinocytes, which indicates that other pathogenic mechanisms might be involved.

### Diagnosis

To our knowledge, there are no reports of cases of hypersensitivity reactions to PZA which were assessed with the currently available *in vitro* tests. As far as skin testing is concerned, in a subject who had experienced an anaphylactic reaction to PZA, an IgE-mediated mechanism was diagnosed by Bavbek et al [127] on the basis of a positive SPT at a concentration of 500 mg/mL in normal saline.

In some cases of immediate skin rashes after the first dose of PZA, hypersensitivity was diagnosed by re-administering PZA [120–126]. It is interesting to note that Mulliez et al did not observe any increase of serum tryptase or urinary histamine in a patient who reacted to PZA re-administration [125].

A slower PZA re-administration has been successful in a few cases of PZA hypersensitivity [122–125], including the one with a positive prick test [127]. In conclusion, PZA hypersensitivity should be suspected if an immediate skin rash develops at initiation of an antituberculous therapy.

### Management

In subjects with cutaneous hypersensitivity reactions during antituberculous therapy, if the skin involvement is not severe, sequential reintroduction of the suspected drugs first at low, then at full dosage should be attempted. PZA should be reintroduced last and at a dose lower than the full therapeutic one, and then administered with a stepwise dose increment.

## Quinolones

### Introductory remarks

Quinolones are usually well-tolerated antibiotics that are being increasingly prescribed because of their effectiveness against Gram-positive and Gram-negative bacteria. These are particularly used in aged populations with the subsequent risks of severe reactions like anaphylaxis.

### Epidemiology and risk factors

The frequency of hypersensitivity reactions to quinolones seems to have increased over the last few years and most of these reactions are of the immediate type [128]. The frequency of anaphylaxis induced by quinolones has been estimated to be 1.8-2.3 per 10,000,000 days of treatment [129]. Moxifloxacin was the quinolone most frequently involved, followed by levofloxacin and ciprofloxacin [130]. The rate of anaphylactic reactions to levofloxacin is reported to be 1 per 1 million patients [131].

Atopy seems to be a risk factor for immediate hypersensitivity [132]. The reactions induced by moxifloxacin were more severe than those induced by ciprofloxacin. In a large group of patients with quinolone hypersensitivity in Spain, 75% of reactions induced by moxifloxacin and 54% of those induced by ciprofloxacin were anaphylactic [133].

### Clinical manifestations

Most hypersensitivity reactions to quinolones are of the immediate type, mainly urticaria and anaphylaxis [128–133]. Nonimmediate reactions are less frequent, and include maculopapular exanthema, fixed drug eruptions, photoallergy, AGEP, SJS, and TEN [128].

### Pathogenesis

In immediate-type reactions, an IgE-mediated mechanism is involved, at least in half of the patients. This was shown by Manfredi et al [132] using radioimmunoassay (RIA), and Aranda et al confirmed that result with RIA and basophil activation test (BAT). When the RIA was repeated after some months, there was a decrease in IgE response, becoming negative in some patients [133].

A T cell–mediated pathomechanism is likely to be involved in exanthematous reactions and phototoxicity [134]. Cross-reactivity is common between first and second-generation quinolones, less common with third and fourth generation quinolones. It is often unpredictable [135, 136]. Immediate hypersensitivity to quinolones has been recently associated with neuromuscular blocking agent sensitization [137].

### Diagnosis

The diagnosis can be difficult because skin testing can induce false positive and false negative results [138]. The former may be explained by the ability of some quinolones to induce direct mast cell histamine release [138]. The concentrations most commonly used for SPT are 5 mg/mL for levofloxacin, 2 mg/mL for ciprofloxacin, and 1.6 mg/mL for moxifloxacin. For intradermal tests there is a wide range of reported concentrations to test with ranging from dilutions 1/1000 to 1/100 [33, 128, 138], with some investigators being unable to find non-irritating concentrations [128, 138, 139]. Radioimmunoassay and BAT have been used, but are not widely available yet. BAT, if negative for the culprit quinolone, is a valuable tool in the decision whether or not to perform provocation tests (DPT) [140]. The provocation test remains the gold standard in diagnosis, despite the risks involved.

Patch tests have had inconsistent and conflicting results in the diagnosis of non-immediate reactions to quinolones [125].

### Management

Avoidance of the group is usually advised, but a careful allergy workout with skin tests, RIA and/or BAT if available, and DPT can confirm tolerance in almost 90% of patients evaluated [128, 140]. Desensitization protocols have also been reported for ciprofloxacin [141, 142].

## Rifampicin

### Introductory remarks

Allergic reactions to rifampicin were recognized soon after its introduction, including fever, flu-like syndrome, rash, thrombocytopenia, acute renal failure, urticaria, and anaphylactic syndrome.

### Epidemiology and risk factors

The incidence of adverse reactions to Rifampicin is variable according to different studies. Hepatitis is one of the common adverse reactions to rifampicin that occurs within the initial few weeks of treatment. A meta-analysis showed that 2.6% of patients who took isoniazid and rifampicin concomitantly and 1.1% of patients who took rifampicin exclusively developed hepatitis [143]. Asymptomatic mild elevation of transaminases (<5 times the upper limit of normal) is common during anti-tuberculosis treatment and rifampicin can be continued without disruption.

### Pathogenesis

Allergic reactions to rifampicin are mediated by different immune mechanisms [144]. Urticaria and anaphylaxis are mediated by type 1 (IgE-mediated) responses [145], and anti-rifampicin IgE in patients’ serum has been demonstrated by CAP and intradermal skin tests done at the concentration of 0.006 mg/mL [146, 147]. Acute renal failure, thrombocytopenia and hemolytic anemia may be mediated by type 2 (antibody-dependent cytotoxicity) responses. Flu-like syndrome or serum sickness has been proposed to be due to type 3 (immune complex) responses. Hypersensitivity reactions may be involved in the pathogenesis of rifampicin-induced hepatitis but other mechanisms such as oxidative metabolism, genotypes of cytochrome p450 2E1, glutathione S-transferase M1, and N-acetyl transferase may also be involved [148].

### Clinical manifestations

Cutaneous reactions are the most common adverse reactions induced by rifampicin and this drug is a frequent culprit of skin reactions during anti-tuberculosis treatment. Flushing affecting the face and neck are common and usually transient. Flu-like syndrome usually begins within 1-2 hours after each administration; the incidence is much higher when rifampicin is intermittently administered, and the majority of patients showing flu-like syndrome will tolerate rifampicin if administered daily [89, 149]. Other cutaneous manifestations include maculopapular rashes, pemphigus, lupus erythematous, SJS/TEN, urticaria, and anaphylaxis [89].

Tuberculosis is more prevalent in HIV-positive patients, and HIV infection is a well known risk factor for drug allergy. Thrombocytopenia is the most common hematological adverse reaction and is associated with anti-rifampicin antibodies that bind to platelet membrane GP1b/IX complex [150]. Rifampicin can induce various renal toxicities, including acute tubular necrosis, rapid progressive glomerulonephritis, acute interstitial nephritis, minimal change nephrotic syndrome and light chain proteinuria [151]. The adverse reactions are usually dose-dependent, and some of them occur more frequently when the drug is administered intermittently. Girling divided the adverse reactions to rifampicin into 2 categories. One group is composed of reactions that occur with daily administration, such as skin rashes, gastrointestinal effects, hepatitis and thrombocytopenia. The second group is constituted by reactions that occur only with intermittent administration such as flu-like syndrome, hemolytic anemia, acute renal failure and anaphylaxis [152].

### Diagnosis

Intradermal skin tests with a 1:10,000 dilution, which has been shown to be non-irritative, have been recommended for the diagnosis of immediate urticarial reactions to rifampicin [153].

### Management

For patients with rifampicin allergy various desensitization protocols have been published [97, 154]. As rifampicin is one of the key drugs for first line anti-TB treatment and it is essential for treatment of isoniazid resistant tuberculosis, desensitization or graded challenge is frequently required in practice. The Japanese Society for Tuberculosis recommended a desensitization protocol which starts from 25 mg, a dose that is usually recommended at graded challenge, and requires 16 days to reach the maintenance dose [154]. A multicenter retrospective study proved the efficacy of this graded challenge procedure [155]. Other authors reported a rapid desensitization protocol which starts from 0.1 mg and requires 6-11 hours to achieve the maintenance dose [97, 156]. In case of serious adverse reactions such as thrombocytopenia, hemolytic anemia or acute renal failure desensitization or graded challenge treatment are contraindicated and rifampicin should be discontinued permanently.

## Streptomycin

### Introductory remarks

Although already mentioned in the Aminoglycoside section, Streptomycin still remains as one of the active treatment agents for Mycobacterium tuberculosis, non-tuberculous Mycobacteria, and brucellosis. Therefore, a separate section on Streptomycin has been considered pertinent.

### Epidemiology and risk factors

About 1-5% of patients may experience hypersensitivity reactions. Patients may develop anaphylaxis at a trivial dose of streptomycin. Exposure to streptomycin through an impaired skin barrier [30], or during oocyte retrieval procedure for artificial fertilization [31] can induce anaphylaxis. Even skin prick test can induce anaphylaxis, so SPT should be carried out at a low starting concentration such as from 1 ng/mL [30, 31].

### Clinical manifestations

Streptomycin can induce skin rash, anaphylaxis, ototoxicity and nephrotoxicity. Streptomycin is usually administered as a daily low dose (15 mg/kg/day) or intermittent high dose (25 mg/kg 3 times per week) for treatment of tuberculosis and the incidence of ototoxicity and nephrotoxicity are no different when using those two protocols [157]. Ototoxicity is usually dose-dependent with vestibular damage and vertigo. The majority of the vestibular toxicities are transient. However, hearing loss from cochlear toxicity may occur and damage may persist despite drug discontinuation [157]. Streptomycin can induce renal damage, such as acute tubular necrosis, in 0.1-1% of patients with tuberculosis and it usually recovers after discontinuation.

Various additional skin reactions to streptomycin have been reported including maculopapular exanthema, erythema, urticaria, exfoliative dermatitis, SJS and DiHS [89].

### Pathogenesis

There is no evidence of streptomycin-specific IgE involved in reactions to this antibiotic. In patients with haemolytic anemia antistreptomycin IgG antibodies have been demonstrated by means of Coombs test.

### Diagnosis

Skin tests are not validated, but positive reactions have been observed [27, 31]. For cell-mediated reactions to streptomycin, patch tests with a 20% drug concentration in petrolatum have been suggested [27, 32].

### Management

In patients with streptomycin allergy avoidance of the antibiotic is recommended. Cross-reactions between neomycin, netilmycin, and streptomycin are observed in 1 to 5% of the cases. A 3-hour desensitization protocol with streptomycin beginning with 1 mg administered intravenously has been proposed [27, 38, 39].

## Sulphamethoxazole – trimethoprim (cotrimoxazole)

### Introductory remarks

**Figure 1:**
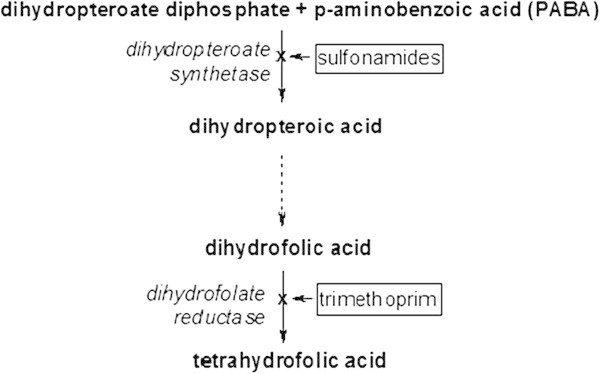
**Effect of cotrimoxazole (Sulfamethoxazole and Trimethoprim) on folate synthesis.**

The combination of the two anti-infectious agents, sulfamethoxazole (SMX) and trimethoprim (TRP), is called cotrimoxazole and is marketed worldwide as Bactrim®, Septra®, Cotrim® etc. The combination has been claimed to be superior to each single agent alone, as TRP and SMX together inhibit successive steps in the folate synthesis pathway (see Figure 1) [158, 159]. It is used to treat bacterial urinary tract infections, otitis media, bronchitis, skin and wound infections, traveler's diarrhea, shigellosis and other infections caused by sensitive organisms. It is also used to prevent or treat *Pneumocystis Jiroveci* pneumonia, toxoplasmosis and nocardiosis in immune-suppressed patients [159, 160] and is an established therapeutic option to prevent relapses in locoregional granulomatosis with polyangiitis [161]. Cotrimoxazole should not be used in children less than 2 months of age due to the risk of serious side effects (kernicterus). For the same reason, they are generally contra-indicated in women prior to delivery, and in breast-feeding mothers. The usual recommended Cotrimoxazole dosage in adults is 800 mg SMX/160 mg TRP every 12 hours.

### Epidemiology and risk factors

Cotrimoxazole has been associated with many side effects [162]. The majority are thought to be due to SMX, and only rarely to TRP (Table 1). The majority of patients allergic to sulphamethoxazole can tolerate in fact trimethoprim but there are few cases of trimethoprim allergy. With regard to presumably allergic side effects, it is estimated that around 2% of treated patients without HIV/AIDS develop a hypersensitivity reaction (HR) [163]. As some of these HR are rather severe [164], the use of cotrimoxazole has decreased substantially, and in some countries therapy with TRP alone has been advocated.

**Table 1:** **Side effects linked to Sulfamethoxazole and Trimethoprim (Cotrimoxazole)**

**Sulfamethoxazole**	
• General side effects	
Nausea, vomiting, anorexia, diarrhoea, hypoglycaemia, hypothyroidism, neurological reactions including aseptic meningitis, ataxia, benign intracranial hypertension, convulsions, dizziness. drowsiness, fatigue, headache, insomnia, mental depression, peripheral or optic neuropathies, psychoses, tinnitus, vertigo, and pancreatitis.	
• Hypersensitivity reactions:	
Skin: Exanthema, pruritus, photosensitivity reactions, exfoliative dermatitis, SJS/TEN, erythema nodosum.	
Systemic: DiHS (with involvement of various organs), Henoch Schönlein purpura, interstitial nephritis (DD crystallisation); anaphylaxis/urticaria.	
Blood: eosinophilia, agranulocytosis, aplastic anaemia, thrombocytopenia, leucopenia, hypothrombinemia, acute haemolytic anemia (glucose-6-phosphate dehydrogenase deficiency)	
**Trimethoprim**	
• General side effects	
Nausea, vomiting, anorexia, diarrhea, thrombocytopenia, megaloblastic anemia, hyperkalemia, rise in serum creatinine.	
• Hypersensitivity reactions:	
Drug-induced liver injury (cholestatic and hepatocellular hepatitis)	
IgE-mediated anaphylaxis	

**Figure 2:**
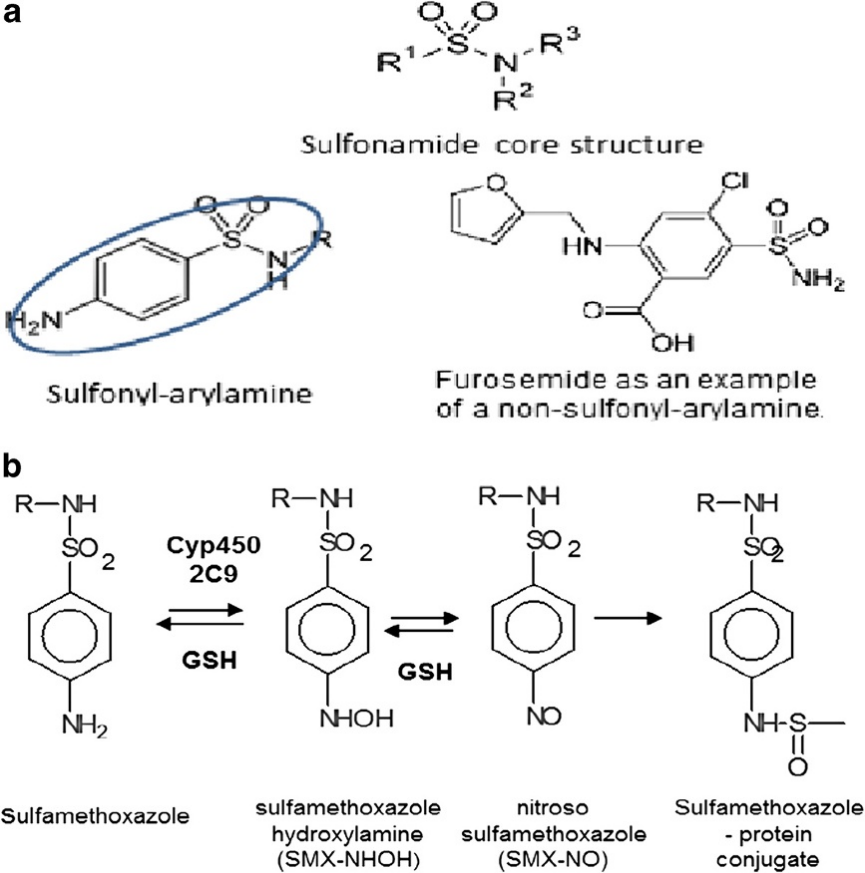
**Sulfonamide chemical structure and metabolism. a**: Sulfonamide core structure. It is present as sulfonyl-arylamine, where a sufonamide is attached to a benzene ring with an unsubstituted amine (-NH2) moiety at the N4 position. Many other drugs may also contain a sulfonamide (example: furosemide). **b**: SMX is metabolized intra-hepatically to SMX-NHOH, which is further oxidised to SMX-NO; the later binds covalently to cystein in proteins.

Different risk factors have been described for cotrimoxazole hypersensitivity :

- HIV infection increases the incidence of allergic side effects and also the intensity of the DHR, as the incidence of SJS/TEN to various drugs including cotrimoxazole is higher in this patient group than in non HIV infected persons [164]. Treatment with cotrimoxazole (3 x weekly twice 800/160 mg) as prophylactic treatment of *Pneumocystis Jiroveci* pneumonia was widely used in patients with acquired immunodeficiency before effective highly active antiretroviral therapy (HAART) was established [165]. Up to 50% of these patients developed skin reactions (mainly “rashes”) of variable intensity. In some patients continuation of therapy was possible (without aggravation); in other patients the therapy had to be stopped.
- Dose and duration of treatment influence the manifestation of drug allergy [166, 167]. It may well be that the shorter and more restricted use of cotrimoxazole, which is nowadays mainly used for 1-3-day treatment of acute cystitis, contributes to the reduced incidence of severe forms of delayed hypersensitivity (DH) to cotrimoxazole.
- Metabolism: the deficiency of glucose-6-phosphatase is a well known risk factor for haemolytic complications with sulphonamide therapy. The metabolism of SMX to reactive compounds (first SMX-NHOH, then SMX-NO) is blocked by glutathione (Figure 2) [168]. The high incidence of “rashes” to cotrimoxazole in HIV + patients had been linked to low glutathione levels in HIV infected patients, but this hypothesis remained controversial [165]. The relationship between slow acetylator phenotype and the manifestation of DH to cotrimoxazole is unclear [169].

### Clinical manifestations

Sulfonamides like SMX, sulfapyrine and sulfadoxine are associated with various side effects, such as nausea, haemopoietic disorders, porphyria, and hypersensitivity reactions. Only some of these adverse effects are mediated by immunological mechanisms and are therefore true allergic reactions.

True allergic reactions of the anaphylactic type (IgE-mediated urticaria and anaphylaxis) are less common [170], as are IgG antibody-mediated reactions (mainly haemolytic anaemia) [171]. The most frequent manifestations of cotrimoxazole hypersensitivity are due to T-cell mediated reactions of varying severity [168]. The most common are “rashes” like maculopapular exanthemas, but sulfonyl-arylamines may potentially induce life threatening reactions like SJS/TEN and DiHS [164, 172]. SJS/TEN appear mainly in the second or third week of treatment. The main features are widespread erythematous macules, flat atypical targets and detachment of the body surface area. The extent of bullous skin lesions is important for the prognosis, as >30% of the most severe cases succumb to this severe DH (TEN). DRESS or *Drug (induced) Hypersensitivity Syndrome* (DHS or DiHS) [172] appears typically after a drug exposure of >2 to 10 weeks. It is clinically characterized by skin rash, fever, lymph node swelling, hepatitis or involvement of other organs (carditis, colitis, pancreatitis, meningitis). Many patients develop facial swelling; some have signs of a capillary leak syndrome, probably related to the excessively high cytokine levels observed during the acute disease. Many patients have activated lymphocytes in the circulation (lymphoblasts) and over 70% (but not all) have marked eosinophilia. Mortality is about 5-10%, and mainly due to liver failure. The clinical course is often complicated. Symptoms may reappear long after stopping the culprit drug. These are often related to reactivation of herpes viruses, in particular human herpes virus 6 (HHV-6), EBV or CMV [173]. Of importance is also the intolerance of other drugs/chemicals during the active phase of DRESS/DiHS, leading to so called flare up reactions [173].

### Pathophysiology of sulfonyl-arylamine allergies

SMX (and the structurally related sulfadoxine, sulfapyrine, a component of sulfasalazine [174], and dapsone, see above) are sulfonyl-arylamines. They are characterized by a sulfonamide moiety directly attached to a benzene ring and an unsubstituted amine (-NH2) at the N4 position (Figure 2a) [174]. The mechanism of sulfonyl-arylamine hypersensitivity reactions involves IgE, occasionally IgG and different types of T-cell mediated reactions [168, 170, 171, 174]. SMX is a pro-drug: it is metabolized intrahepatically (Cytochrome P450 2C9) to SMX-NHOH, which is further oxidised to SMX-NO (Figure 2b) [168]. SMX-NO is highly reactive by binding to cysteins in soluble and cell bound proteins. It thus can elicit an IgE and/or a T cell mediated response to modified proteins which can result in different clinical presentations.

More importantly SMX is able to directly bind to immune receptors. It is a typical example of the *p-i concept* (pharmacological interaction with immune receptor concept), namely that a drug can directly bind to the HLA (p-i HLA) and/or TCR (p-i TCR) and thereby indirectly or directly elicit T-cell stimulation [168, 175].

As immune reactions are directed to the structural component, patients with an allergy to a sulfonyl-arylamine may cross-react with other sulfonyl-arylamines, but not to sulfonamides in general. Laboratory analysis of T cell reactions and clinical data show that non-sulfonyl-arylamine drugs like glibenclamide (glyburide), furosemide, and celecoxib are not stimulatory in patients allergic to sulfonyl-arylamides [168, 174, 176]. The absence of cross-reactivity between sulfonamide antibiotics and non-antibiotics has been shown in large cohorts [163] and withholding non-antibiotic sulfonamides in sulfonamide allergic patients is no longer standard of care.

### Diagnosis

Sulfonamide hypersensitivity reactions are mainly allergies to sulfonyl-arylamines. They can be clinically suspected by the constellation of exposure, timing, patterns of organ manifestations and underlying conditions.

The majority of SMX reactions involve T cells. An allergy workup is normally recommended 1 to 6 months after the reaction. It may comprise skin tests and *in vitro* tests. The sensitivity of these tests is probably low, but the specificity is good – which makes a positive result valuable. IDT may be helpful in both immediate and non-immediate reactions. Sulfamethoxazole at a concentration of 80 mg/mL has been shown to be non-irritating in IDT [33], but the sensitivity of IDT using SMX in different skin manifestations is not known. In addition, IDT have a small risk for eliciting systemic allergic reactions (mostly mild and transient). Patch testing [177] and Lymphocyte Transformation Tests (LTT) [10] are used in Europe in non-immediate reactions. The latter seems to have a fairly good sensitivity and specificity in severe reactions like DiHS but not in SJS/TEN [10, 178].

The risk of patch test (10% in dimethyl sulfoxide or petrolatum) is negligible; however its sensitivity seems to be lower than late (24 hr) reading of intradermal tests [177]. In our experience the LTT seems to be more sensitive and allows also testing compounds *in vitro* which are not available for in vivo tests. However, the LTT and its variants are still rather complex procedures, which require skilled personnel and experience with the drug in *in vitro* assays [10].

### Management

In case of assumed hypersensitivity, the presumably causative drug is generally immediately withdrawn. However, in mild, non-immediate sulfamethoxazole reactions (rashes) without signs of mucosal or extra-cutaneous symptoms, the cotrimoxazole treatment may be continued or re-administered following a “desensitization” protocol. Such “treating through” or “desensitization” is most often used in HIV + patients [178, 179] and is successful in 44.4-79% [180]. It requires monitoring for systemic involvement (fever, eosinophilia, lymphadenopathy, hepatitis). In most cases an immune mediated pathomechanism has not been shown.

In localized mild exanthems stopping the treatment and topical corticosteroids plus an antihistamine for 3-6 days might be sufficient. Patients with sulfonyl-arylamine induced SJS/TEN should be handled like other SJS/TEN patients and be best referred to specialized (e.g. burn-) centres experienced in the care of such patients. In severe non-immediate reactions like DRESS/DiHS the T-cell immune system is massively activated and may temporarily react to many “innocuous” drugs with a flare up [173]. Thus it is our practice to minimize any drug therapy in patients as long as activated lymphocytes are detectable in the circulation. For the treatment of DiHS with severe organ involvements (e.g. ALAT/ASAT values > 500) corticosteroids are often used but not proven as efficacious in studies.

## Telithromycin

### Introductory remarks

Telithromycin, the first antibacterial agent commercialized from the group of ketolides, is a semisynthetic derivative of the 14-membered macrolide erythromycin [181]. Telithromycin differs structurally from macrolides in the substitution of a 3-keto function in place of the L-cladinose moiety. An aromatic N-substituted carbamate extension differentiates erythromycin from clarithromycin. Telithromycin is approved for upper and lower respiratory tract infections [182].

### Epidemiology and risk factors

Although clinical trials and post-marketing studies have only detected mild adverse events [183], the incidence of telithromycin hypersensitivity is not known and the risk factors have not been identified.

### Pathogenesis and clinical manifestations

Considering adverse events, concern exists about telithromycin hepatotoxicity [183]. Of three patients who developed severe hepatotoxicity within a few days of taking telithromycin for upper airway infection, one recovered spontaneously, one required liver transplantation and one died [184, 185]. The histologic examination of two cases showed massive hepatic necrosis.

Hypersensitivity reactions to macrolides are rare, with urticaria and angioedema being the most common symptoms (112,115,183). Descriptions of telithromycin hypersensitivity are anecdotal [186, 187]. A report exists of a life-threatening immediate-type hypersensitivity reaction after the first administration of telithromycin prescribed for an upper respiratory tract infection [186]. Shortly after ingestion of this first dose severe shortness of breath and airway obstruction developed, requiring adrenalin and intubation. This patient had previously tolerated erythromycin and azithromycin. The diagnosis was based on the convincing clinical history and skin testing or drug provocation tests were not done.

Regarding non immediate hypersensitivity reactions, TEN has been reported in a 26-year-old woman who received telithromycin for sinusitis [188]. She had epidermal detachment affecting more than 50% of her total body surface area and received treatment with intravenous immunoglobulins.

### Diagnosis

The diagnosis of telithromycin allergy is based mainly on drug provocation testing, as skin testing is of little use [112, 115, 189].

### Management

Although hypersensitivity reactions to telithromycin are rare they have been reported, including severe hepatotoxicity. These observations need to be considered when prescribing telithromycin.

## Tetracyclines

### Introductory remarks

Tetracyclines are antimicrobial agents that have been in use since 1948. They inhibit protein synthesis by interacting with the bacterial ribosome and therefore these antibiotics are considered bacteriostatic. The chemical structure consists of four tetra- hydrocarbon rings with a “cycl” derivation. They belong to a subclass of polyketide compounds that have an octahydrotetracene-2-carboxamide skeleton. They are collectively known as derivatives of polycyclic naphthacene carboxamide [189]. Classical natural occurring members of this group are: tetracycline, chlortetracycline, oxytetracycline, declocycline and other semisynthetic like doxycycline and minocycline [187]. They are amphoteric antibiotics forming acid or basic salts which are soluble in water. As they are ampholytes, the optimal solubility is observed in basic and alkaline solutions [190].

They are used for treating infectious diseases caused by Gram-negative and Gram-positive bacteria such as pelvic infections, bronchitis, urinary tract infections, as well as infections caused by rickettsia, chlamydia and mycoplasma species. Other indications are acne vulgaris, bullous pemphigus, rosacea and rheumatoid arthritis [189]. They cross the placental barrier and can accumulate in the long tubular bones and the teeth. These antibiotics are contraindicated in children and in women after the fifth week of pregnancy [191].

### Epidemiology, risk factors and clinical manifestations

Amongst the adverse side effects, allergic and autoimmune drug reactions have been reported [192, 193]. In spite of the adverse effects tetracycline has been shown to diminish the rash severity in patients treated with epidermal growth factor receptor (EGFR) inhibitors [194]. They have also been implicated in inhibiting the IgE-mediated responses and in the attenuation of the allergic response by inhibiting the NF-kB pathway [195].

### Pathogenesis

Although classical hypersensitivity reactions are considered much less common than for beta-lactams and other antibiotics, tetracyclines have been implicated in both IgE- [195] and T cell-dependent reactions such as fixed drug eruption [196–199], more severe reactions like DiHS and TEN [200], and reactions involving specific organs such as liver, lungs, and the central nervous system amongst others [200]. Some of these severe reactions have been followed by multiple autoimmune sequelae [199].

Immediate reactions can be anaphylactic when a suspected IgE-mediated mechanism [201] or a non- allergic hypersensitivity reaction are considered [199, 201]. All these reactions occur within a short interval after drug intake and tetracycline [202], doxycycline [203], and minocycline [204] have been implicated. In the case of minocycline an intermediate metabolite has been postulated as the cause of the reaction although further evaluation is required [192].

All tetracyclines share a polycyclic nucleus although with different side chains. They may generate common epitopes responsible for the cross-reactivity or unique structures that elicit specific selective responses to only a single drug within the tetracycline group as occurs with beta-lactam antibiotics [205]. Most of the information concerning cross reactivity has been reported with fixed drug eruptions, the most common hypersensitivity reaction induced by these drugs. Cross reactivity between tetracycline hydrochloride and oxytetracycline has been reported as well as between demethylchlortetracycline, doxicycline and minocycline [192–195, 197, 202]. This pattern of cross reactivity and selective responses may also occur for the other reactions such as DiHS or organ specific reactions. No information is available for immediate reactions.

Typical characteristic adverse effects are phototoxic and photoallergic reactions. These are T cell responses directed to photoadducts which originate in the skin. They usually occur after 5 days of drug administration although they may appear within hours and develop progressively spreading over the skin not exposed to ultraviolet radiation. The most common tetracycline involved in these reactions has been minocycline. Death can occur specially in those patients who develop fulminant hepatitis or respiratory failure [198].

### Diagnosis

General principles recommended for in vivo diagnostic tests can be followed for the diagnosis of hypersensitivity reactions to tetracyclines [6]. These consist on SPT/IDT for immediate reactions and IDT/patch testing for non immediate reactions. For doxycycline, concentrations of 20 mg/mL can be used for SPT and for IDT the maximum non-irritative concentration recommended is one tenth dilution of this (2 mg/mL) [6]. Concentrations above these can induce false positive reactions. Concentrations for patch testing of 5% w/v or w/w in petrolatum have been recommended. In the photopatch test the drug is applied on the back using an aluminum chamber and 48 hours later irradiation with a UVA lamp is made with a dose of 10 jls/cm2. General recommendations are available for this procedure [206]. Photopatch tests with doxycycline in appropriate dilution are useful to confirm photoallergic reactions to this antibiotic [207].

### Management

In cases of immediate hypersensitivity reactions to tetracyclines these drugs must be withdrawn from the treatment and substituted with alternative drugs with similar antibacterial spectrum. In view of the lack of sufficient information on whether selective responses or cross-reactivity occurs, this is the most conservative and safest approach that can be recommended.

## Vancomycin

### Introductory remarks

Vancomycin, a glycopeptide, has been often used in infections with beta-lactam resistant Gram-positive organisms or in beta-lactam allergic patients. Its use continues to rise with the spread of community and hospital-acquired methicillin-resistant *Staphylococcus aureus* as well as in persistent and moderate-to-severe cases of *Clostridium difficile* colitis.

### Epidemiology and risk factors

The most common hypersensitivity reaction associated with vancomycin is the *red man syndrome* (RMS). The incidence varies between 3.7 and 47% in infected patients and between 30 and 90% in healthy volunteers who received this antibiotic [208, 209]. Most severe reactions occur in patients younger than the age of 40, particularly in children. Mastocytosis, as well as the use of agents that activate mast cells, such as opioids, muscle relaxants, and radiocontrast media, can increase the risk of developing RMS upon the infusion of vancomycin [210, 211].

Immune-mediated immediate and non-immediate reactions to vancomycin seem to be infrequent. IgE-mediated anaphylaxis due to vancomycin is believed to be rare, although reactions with demonstrable drug-specific IgE have been described [212–214]. Other reactions, including hematologic and renal disorders, drug fever, and phlebitis, can also occur but are uncommon [215, 216].

### Clinical manifestations

Vancomycin can elicit a large variety of hypersensitivity reactions, ranging from localized skin reactions to generalized cardiovascular collapse. However, the most frequent immediate hypersensitivity is the RMS, characterized by flushing, warmth, pruritus, and hypotension. RMS is a rate-dependent infusion reaction. Pain, muscle spasms in the back and chest and dyspnea may also occur. RMS is rarely life-threatening, but severe cardiovascular toxicity has been reported. Angioedema, wheezing and respiratory distress are more common in anaphylaxis than in severe RMS, whereas RMS more typically presents with chest pain causing a sensation of chest tightness [217–220].

Vancomycin can elicit a variety of non-immediate cutaneous and systemic reactions. SJS, exfoliative dermatitis, TEN, extensive drug fixed eruption, and leukocytoclastic vasculitis have all been described in association with vancomycin use in case reports. Other forms of hypersensitivity include DiHS syndrome/DRESS, AGEP, linear IgA bullous dermatosis and renal disorders [215, 221–226].

### Pathogenesis

Vancomycin is responsible for several different types of immunological and non-immunological hypersensitivity reactions (Table 2). The RMS results from direct mast cell and basophil histamine release, can occur without prior exposure, and is not commonly accompanied by an increase in serum tryptase [227].

**Table 2:** **Mechanisms and manifestations of Vancomycin hypersensitivity**

Mechanisms		Type of reaction based on the time of onset	Main manifestations due to vancomycin
**Non-immunological mechanism**	**Mast cell and basophil histamine release**	**Immediate reaction**	**Red man syndrome**
**Immunological mechanisms**	**Type I**	**Immediate reaction**	**Anaphylaxis**
	**Type II**	**Non-immediate reactions**	**Nephritis**
	**Type III**	**Non-immediate reactions**	Vasculitis*
	**Type IVa**	**Non-immediate reactions**	**Exfoliative dermatitis**
	**Type IVb**	**Non-immediate reactions**	**Maculo-papular exanthema, DRESS/DiHS**
	**Type IVc**	**Non-immediate reactions**	**SJS/TEN, hepatitis**
	**Type IVd**	**Non-immediate reactions**	**AGEP**

*Unproven.

### Diagnosis

A detailed clinical history supports an appropriate diagnosis and allows the distinction between immediate and non-immediate reactions. Different from RMS, IgE-mediated anaphylaxis usually does not occur on the first administration of the medication. Patients with anaphylactic reactions to vancomycin often have a history of multiple prior exposures.

*In vitro* tests are generally not useful in the diagnosis of vancomycin reactions. Elevated plasma histamine and tryptase may be found in severe RMS, and therefore are not useful in differentiating RMS from anaphylaxis based on these levels [227].

Although the value of skin tests appears to be uncertain and false-positive reactions may occur when the antibiotic is tested in high concentrations, we consider that the skin testing with appropriate vancomycin concentrations may reflect clinical reactivity and provide supportive evidence for clinical features. A positive skin test (intradermal skin test) at concentrations of 0.1 mg/mL or lower is suggestive of drug allergy in the setting of an appropriate clinical history [227].

Positive vancomycin patch test at a concentration of 0.005% in water has been described in non-immediate reactions [228].

### Management

The early recognition and discontinuation of the drug are critical. Severe RMS can mimic IgE-mediated anaphylaxis and requires immediate diagnosis and management [229].

In contrast to true allergic hypersensitivity reactions, slowing the infusion rate of vancomycin to 500 mg given over one hour usually reduces the chance of developing RMS. There are few studies regarding the effectiveness of antihistamines as premedication to prevent RMS; therefore, the empiric use of premedication should be avoided except for cases in which more rapid infusion of vancomycin (rates exceeding 1 g over one hour) are necessary. In these cases, the combined use of H1 and H2 antihistamines is recommended one hour before the infusion [209, 230–232].

Various series have been published on successful vancomycin desensitization regimes, both rapid (over hours) and slow (over days); in particular in patients with opioids treatment a very successful desensitization protocol has been used permitting pain control while treating beta-lactam resistant severe infections [233]. Effective desensitization regimes have been described in the treatment of vancomycin anaphylaxis. Desensitization to vancomycin has been successfully performed for both suspected IgE-mediated reactions and for severe immediate RMS refractory to pre-medication [208, 213, 234].

Teicoplanin, another glycopeptide with the same spectrum of antimicrobial activity, has fewer side effects compared to vancomycin. Although there have been reports of cross-reactivity in individuals with vancomycin and teicoplanin allergy, there have also been reports of patients with teicoplanin allergy who tolerated vancomycin. RMS is very unusual with teicoplanin and immediate reactions and non-immediate reactions are infrequent [235].

## Concluding remarks

Adverse reactions to non beta-lactam antimicrobials are relatively frequent. Physicians should be informed on the potential risks of these medications, and the management of untoward manifestations when they occur. In Table 3 a summary of most common reactions and suggested diagnostic tests are presented.

**Table 3:** Adverse reactions to non beta-lactam antimicrobials: clinical picture and diagnostic tests*

Drugs	Clinical manifestations	Diagnostic tests	Drug concentrations
**Aminoglycosides**	CD, U, MPE, FDE, TEN, DIHS, ANA	PT	1-20%
		STs	0.1 ng/mL-20 mg/mL
**Chloramphenicol**	ANA, fever, U, AE, MPE, AGEP, CD, bullous eruption, EM, FDE, SJS, TEN, bronchospasm, aplastic anemia	STs	-
		PT	1%
**Clindamycin**	MPE, ANA, U, AE, FDE, bullous eruptions, AGEP, Sweet’s syndrome, SJS, DIHS	OPT	-
		PT	10%
**Dapsone**	Methemoglobinemia, agranulocytosis	Methemoglobin measurement	-
**Ethambutol**	Skin rash, ED, U, AE, EM, SJS, TEN, blood eosinophilia, pulmonary infiltrates, hepatitis, vomiting, thrombocytopenia, flu-like syndrome, arthralgia, neuropsychiatric symptoms, optic neuritis	PT	10-50%
**Isoniazid**	MPE, lichenoid rash, flushing, DIHS, SJS, pruritus, drug fever, peripheral neuropathy, hepatitis	Graded challenge	-
**Macrolides**	U, AE, RC, ANA, MPE, CD, FDE, TEN	STs	Erythromycin 0.05 mg/mL
			Azithromycin 0.01 mg/mL
			Roxythromycin 50 mg/mL
			Clarithromycin 0.5 mg/mL
		PT	10%
		OPT	-
**Pyrazinamide**	Flushing, itchy rash, ANA, hepatitis	STs	500 mg/mL
**Quinolones**	U, ANA, MPE, FDE, photoallergy, AGEP, SJS, TEN	STs	Levofloxacin 5 mg/mL
			Ciprofloxacin 2 mg/mL
			Moxifloxacin 1.6 mg/mL
		OPT	
**Rifampicin**	Flushing, flu-like syndrome, MPE, pemphigus, lupus erythematosus, SJS, TEN, ANA, thrombocytopenia, nephrotoxicity, hepatitis, hemolytic anemia	STs	1: 10000
**Streptomycin**	Skin rash, ANA, ototoxicity, nephrotoxicity, MPE, U, ED, SJS, DIHS	PT	20%
**Sulphamethoxazole**	Nausea, haemopoietic disorders, porphyria, ANA, haemolyitc anemia, MPE, SJS, TEN, DIHS	STs	80 mg/mL
		PT	10%
		LTT	
**Telithromycin**	Hepatotoxicity, U, AE, ANA, TEN	OPT	-
**Tetracyclines**	FDE, DIHS, TEN, hepatitis, pneumonitis, ANA, phototoxic and photallergic reactions	STs	Doxycicline :prick 20 mg/mL; id: 2 mg/mL
		PT	5%
**Vancomycin**	RMS, ANA, SJS, ED, TEN, FDE, vasculitis, DIHS, AGEP, linear IgA bullous dermatosis, nephropathy	STs	0.1 mg/mL
		PT	0.005%

*Concentrations given are drawn from the literature. Many of those have not been validated.

**Legend:** ANA: anaphylaxis; AE: angioedema; CD: contact dermatitis; DIHS: drug-induced hypersensitivity syndrome (DRESS); EM: erythema multiforme; ED: exfoliative dermatitis; FDE: fixed drug eruption; LTT: lymphocyte transformation test; MPE: maculopapular exanthema; OPT: oral provocation test; PT: patch tests; RMS: red man syndrome; RC: rhinoconjunctivitis; SJS: Stevens-Johnson syndrome; STs: skin tests; TEN: toxic epidermal necrolysis; U: urticaria.
